# Successful Fluid Management in Respiratory Failure due to Clazosentan Following a Cerebral Aneurysm Clipping: A Case Report

**DOI:** 10.7759/cureus.54850

**Published:** 2024-02-25

**Authors:** Hirotaka Kinoshita, Kodai Kato, Yuma Yamazaki, Eiji Hashiba, Kazuyoshi Hirota

**Affiliations:** 1 Department of Anesthesiology, Hirosaki University Graduate School of Medicine, Hirosaki, JPN; 2 Division of Intensive Care, Hirosaki University Hospital, Hirosaki, JPN

**Keywords:** hypernatremia, pulmonary edema, hemodynamic system, respiratory failure, clazosentan

## Abstract

Clazosentan, a potent selective endothelin receptor subtype A antagonist, has been demonstrated to be effective in preventing cerebral vasospasms after subarachnoid hemorrhage. We report the successful management of respiratory failure due to pulmonary edema associated with clazosentan, with a hemodynamic monitoring system. A 49-year-old Japanese man underwent emergency clipping for a right internal carotid-posterior communicating artery aneurysm. The surgery and general anesthesia for the rupture proceeded with no complications. Clazosentan was administered from postoperative day 1 to prevent cerebral vasospasm. He presented with respiratory failure six days post surgery and chest X-ray imaging showed pulmonary edema. In our intensive care unit, the patient's N-terminal pro-brain natriuretic peptide was 476 pg/mL although trans-thoracic echography indicated a normal left ventricular ejection fraction (>60%) and normal diastolic function. The hemodynamic monitoring system showed 11 L/minute cardiac output and a cardiac index of 5.6 L/minute/m^2^. We thus diagnosed the cause of the patient's respiratory failure as due to excessive volume, as an adverse event of clazosentan. We changed the cerebral vasospasm-preventive drug to fasudil hydrochloride hydrate and forced urination. His body weight dropped approximately 9 kg as of day 9 in the ICU and he was weaned off the ventilator 23 days post surgery. This case indicates the importance of optimal infusion in patients with clazosentan. Optimal fluid management using a hemodynamic monitoring system could be useful for clazosentan-induced respiratory failure.

## Introduction

After an individual experiences a subarachnoid hemorrhage (SAH), a cerebral vasospasm can cause delayed cerebral ischemia (DCI), and it is therefore important to prevent such cerebral vasospasms [1]. Clazosentan, a potent selective endothelin receptor subtype A antagonist, has been demonstrated to be effective in preventing cerebral vasospasms after SAH and was approved in Japan for this clinical use in 2022 [2]. However, the vascular permeability that is a result of clazosentan treatment leads to intravascular dehydration [3], and clinicians must therefore pay close attention to the optimal infusion of patients who are treated with clazosentan. In addition, pulmonary edema and pleural effusion are known to be major adverse events of clazosentan therapy [4]. Here, we provide the case details of the successful fluid management during a patient's respiratory failure due to pulmonary edema that was associated with his clazosentan treatment, with our use of a hemodynamic monitoring system.

## Case presentation

A 49-year-old Japanese man (height 171 cm, weight 74 kg) was taken to the hospital with impaired consciousness, and diagnosed with the rupture of a right internal carotid-posterior communicating artery aneurysm. His past medical history was unremarkable. The emergency clipping and general anesthesia for the rupture proceeded with no complications. The patient’s attending neurosurgeon administered clazosentan (300 mg/day) from postoperative day 1 to prevent cerebral vasospasm after confirming on imaging that there was no pleural effusion or pulmonary edema. They also prescribed oral fludrocortisone (0.1 mg/day) as prophylaxis for cerebral salt-wasting syndrome, beginning the same day. He was febrile, and he received large amounts of fluids daily for hypotension during his time in our hospital’s stroke care unit (Table 1).

**Table 1:** Treatment progress in the stroke care unit

Postoperative days	1	2	3	4	5	6
Body weight (kg)	74.8	75.1	74.4	74.6	75	81.6
Urine volume (ml)	3750	2900	3100	2200	2150	1800
Balance (ml)	+1375	+3226	+1174	+2666	+4843	+4101
Sodium (mmol/L)	145	157	162	170	168	165

The patient's oxygenation capacity decreased gradually from the early postoperative period and six days post surgery, he presented with respiratory failure. Upon his subsequent admission to our hospital’s intensive care unit (ICU), his body weight had increased to 81.6 kg, and the patient presented respiratory failure with partial pressure of oxygen (PaO2)/fraction of inspired oxygen (FiO2) ratio, 66, mode, synchronized intermittent mandatory ventilation and pressure support, FIO2, 1.0, pressure of control/pressure of support, 12/12 cmH20, and positive end-expiratory pressure (PEEP), 15 cmH20. The patient’s other vital signs were as follows: heart rate 94 beats per minute, blood pressure 135/54 mmHg with the administration of 0.1 μg/kg/minute noradrenaline, and Glasgow Coma Scale, E3V4M5.

**Figure 1:**
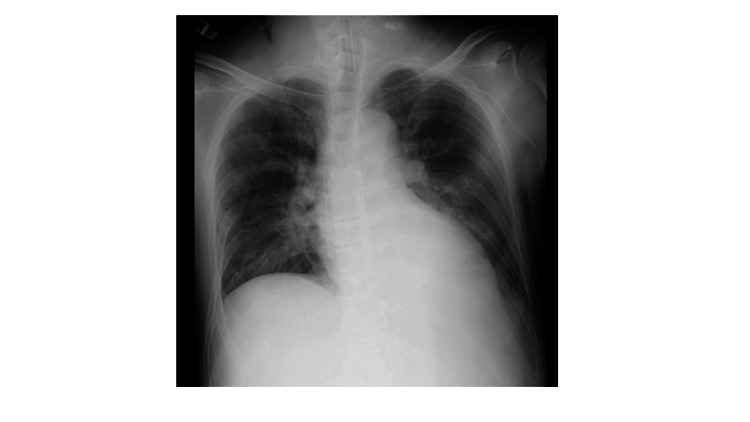
Chest X-ray upon admission to the ICU A chest X-ray examination demonstrated butterfly shadow, heart enlargement, and a positive diaphragm silhouette sign. This suggested the existence of pulmonary edema and pleura effusion.

**Figure 2:**
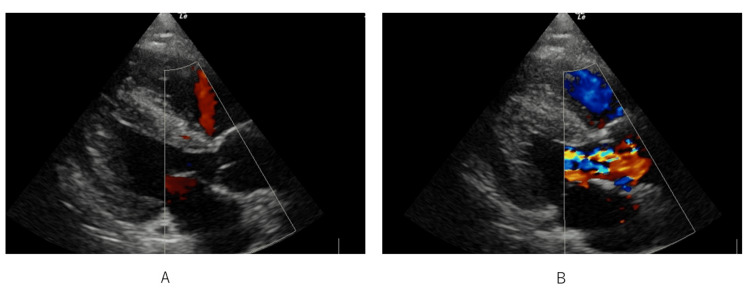
Trans-thoracic echocardiography upon admission to the ICU Trans-thoracic echography indicated a normal left ventricular ejection fraction (>60%) and normal diastolic function. A small amount of pericardial effusion was confirmed. A: Diastolic phase. B: Systolic phase.

A chest X-ray examination demonstrated butterfly shadow, heart enlargement, and a positive diaphragm silhouette sign (Figure 1). The patient’s N-terminal pro-brain natriuretic peptide was 476 pg/mL although trans-thoracic echography indicated a normal left ventricular ejection fraction (>60%) and normal diastolic function (Figure 2). We used a hemodynamic monitoring system, i.e., a LiDCO rapid V3™ (Masimo Corporation, Irvine, California, United States), which showed 11 L/minute cardiac output (CO) and a cardiac index (CI) of 5.6 L/minute/m2. We thus diagnosed the cause of the patient’s respiratory failure as due to excessive volume, as an adverse event of clazosentan. In addition, we considered fludrocortisone as one of the causative agents for fluid overload. 

**Figure 3:**
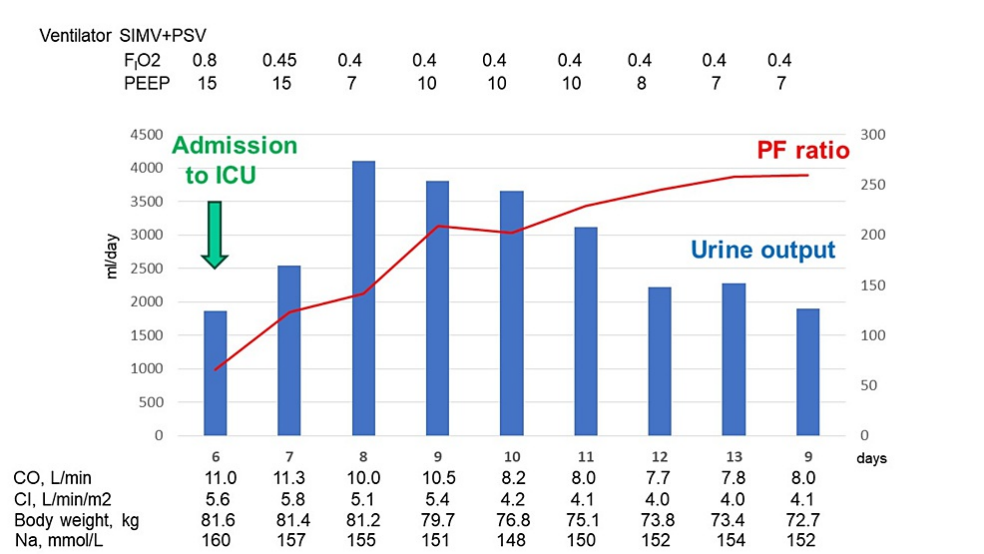
The patient's progress in the intensive care unit. SIMV: synchronized intermittent mandatory ventilation; PS: pressure support ventilation; PEEP: positive end-expiratory pressure; PF ratio: PaO2/FIO2 ratio; CI: cardiac index; CO: cardiac output

We also changed the drug for preventing cerebral vasospasm from clazosentan to fasudil hydrochloride hydrate (90 mg/day). A 165 mmol/L sodium level was observed, and we thus discontinued the patient's fludrocortisone. We administered ventilator respiratory management and hemodynamic therapy with reference to the patient's CO and CI provided by the LiDCOrapid V3™ monitor. His fluid overload and oxygenation capacity improved with the administration of diuretics and high-PEEP ventilator management, as depicted in Figure 3. At that time, the patient's body weight had decreased from 81.6 to 72.7 kg by the ninth day in the ICU.

On the patient's 10th day in the ICU, he was transferred to the stroke care unit. At 23 days post surgery, he was weaned off the ventilator, and a week later he was discharged.

## Discussion

With the use of a hemodynamic monitoring system, we were able to successfully manage this patient's respiratory failure due to pulmonary edema associated with clazosentan treatment. This case highlights the importance of optimal infusion in patients under treatment with clazosentan.

Approximately two-thirds of individuals who experience an SAH also experience a cerebral vasospasm, and among them only half will be clinically symptomatic [5]. The conventional treatments for cerebral vasospasm have focused on flow viscosity, vascular resistance, and blood pressure in the cerebral vasculature. The use of hypervolemia, hemodilution, and hypertension measures is known as 'triple H' therapy. In order to improve neurological outcomes, the calcium channel blocker nimodipine has been used globally in attempts to prevent brain damage caused by reduced blood flow to the brain, and a variety of drugs such as fasudil hydrochloride (a selective ROCK inhibitor) and sodium ozagrel (an antiplatelet agent and a thromboxane A2 synthesis inhibitor) as monotherapy or in combination have been applied to prevent vasospasm [6,7].

A key mediator of vasospasm after SAH is thought to be endothelin, which has a potent, long-lasting vasoconstrictor effect [8]. In an SAH, the release of oxyhemoglobin-derived endothelin from red blood cells is thought to make a significant contribution to the development of cerebral vasospasm due to this vasoconstriction effect [9]. As a selective endothelin receptor subtype A antagonist, clazosentan inhibits endothelin-mediated cerebral vasospasms.

However, treatment with clazosentan was demonstrated in two randomized phase III trials to be associated with several potential adverse events in Japanese patients, including pulmonary edema (11.5 %), pleural effusion (16.9%), and hypotension (3.8%) [4]. A 2023 meta-analysis revealed that clazosentan decreased the risks of vasospasm-related DCI and angiographic vasospasm but did not improve the patients' functional outcomes or mortality; however, the rate of adverse events was increased by clazosentan therapy [10].

Endothelin receptor antagonists, particularly endothelin A-selective antagonists, favor edema formation by activating endothelin B receptors. The underlying mechanisms of this are: (1) fluid retention resulting from the activations of arginine vasopressin and aldosterone that occur secondary to vasodilation, and (2) increased vascular permeability [3]. Up to 75% of patients with SAH experience fever, and patients with SAH and fever tend to have a worse prognosis [11]. The massive infusion of fluids in this febrile patient who was treated with clazosentan led to his respiratory failure. In addition, hypernatremia due to fludrocortisone treatment could be one of the causes of fluid overload. 

A proposed infusion model in critical care is the ROSE (resuscitation, optimization, stabilization, and evacuation) model [12]. When clinicians perform infusions, they must always consider the possibility of an overload after the patient's initial resuscitation. We were able to diagnose the present patient's fluid overload on his admission to the ICU based on the combination of hemodynamic monitoring, echocardiographic findings, and the patient's weight gain. Indeed, his case highlights the importance of fluid monitoring when clazosentan is used.

## Conclusions

We were able to successfully manage an adult male's pulmonary edema and respiratory failure that were associated with his clazosentan regimen. This case indicates the importance of optimal infusion in patients with clazosentan. Optimal fluid management using a hemodynamic monitoring system could be useful for clazosentan-induced respiratory failure. It might be difficult to determine the optimal infusion volume in patients who are being treated with clazosentan, but the present case suggests that beneficial fluid management can be achieved with the use of a hemodynamic monitoring system.
